# Integrating cervical vertebral maturation and spheno-occipital synchondrosis on CT: a non-linear framework for probabilistic age estimation

**DOI:** 10.1007/s00414-026-03752-x

**Published:** 2026-03-02

**Authors:** Gokmen Karabag, Mustafa Bozdag, Ali Er, Eric Baccino, Oguzhan Ekizoglu, Laurent Martrille

**Affiliations:** 1 Faculty of Medicine, Department of Forensic Medicine, Manisa Celal Bayar University, Manisa, Turkey; 2 Department of Radiology, Tepecik Training and Research Hospital, Izmir, Turkey; 3 L’Équipe de Droit Pénal et de Sciences Forensiques de Montpellier (EDPFM), Department of Forensic Medicine, University of Montpellier, CHU Montpellier, Montpellier, F-34000 France; 4 Department of Forensic Medicine, Tepecik Training and Research Hospital, Izmir, Turkey

**Keywords:** Forensic age estimation, Cervical vertebral maturation, Spheno-occipital synchondrosis, Computed tomography, Probabilistic age assessment, Legal age thresholds

## Abstract

The aim of this study was to compare the developmental timing and variability of cervical vertebral maturation (C2–C4) and spheno-occipital synchondrosis (SOS) ossification, and to evaluate these indicators using non-linear growth modelling and probabilistic age-threshold analysis to improve transparent quantification of uncertainty at legally relevant age thresholds (≥ 14, ≥ 16, and ≥ 18 years). This retrospective cross-sectional study analysed CT examinations of 986 individuals aged 10–28 years. Cervical vertebral maturation (C2–C4) and SOS ossification were assessed using five-stage ordinal systems, with age treated as a continuous variable. Skeletal maturation was examined using descriptive analyses, transition analysis, non-linear Gompertz modelling, and probabilistic age-threshold estimation. Machine learning analyses were included for comparative purposes only. Cervical vertebral maturation progressed earlier and with less variability than SOS ossification, with complete fusion most frequently observed in C2, followed by C3 and C4. SOS showed prolonged and heterogeneous maturation with substantial overlap between advanced stages. Non-linear models demonstrated earlier inflection points for cervical vertebrae (approximately 15.8–16.6 years) than for SOS (approximately 18.9 years). Advanced cervical stages were associated with high probabilities of exceeding legal age thresholds, whereas complete SOS fusion alone showed lower discriminative value, particularly for ≥ 18 years. Combined assessment yielded the highest conditional probabilities and reduced uncertainty. Machine learning results were consistent with probabilistic modelling without providing additional biologically interpretable information. Cervical vertebral maturation and SOS ossification represent distinct but complementary aspects of skeletal development. Modelling these indicators as continuous, non-linear, and probabilistic processes enables explicit management of uncertainty and improves the forensic applicability of radiological age estimation, particularly near legally relevant age thresholds.

## Introduction

Age estimation is a fundamental task in forensic medicine and forensic anthropology, underpinning a wide range of legal, civil, and administrative decisions. In both living individuals and skeletal remains, determining whether a person has reached specific legal age thresholds—most notably 14, 16, and 18 years—can directly influence criminal responsibility, asylum procedures, consent, and access to age-dependent rights [1–4]. In such contexts, forensic age assessment must ensure not only scientific accuracy but also transparency, biological plausibility, and explicit acknowledgment of uncertainty.

Radiological evaluation of skeletal maturation has become central to contemporary forensic age estimation due to its non-invasive nature, reproducibility, and capacity to visualise internal skeletal structures in vivo [5]. Among the anatomical regions proposed for this purpose, the cervical vertebrae and the spheno-occipital synchondrosis (SOS) have attracted particular attention, as their maturation spans late childhood, adolescence, and early adulthood—precisely the age range encompassing most legally relevant thresholds [6–8].

The cervical vertebrae, particularly C2–C4, undergo a progressive and relatively ordered maturation process characterised by morphological changes and epiphyseal fusion that generally occur earlier in adolescence [9–15]. In contrast, the spheno-occipital synchondrosis, located between the basiocciput and basisphenoid, represents one of the final growth centres of the cranial base to ossify. The spheno-occipital synchondrosis (SOS) closure is known to display a prolonged transitional phase with considerable inter-individual variability, often extending into early adulthood [7, 8, 16–18]. These distinct biological trajectories suggest that cervical vertebral maturation and SOS ossification provide complementary, rather than interchangeable, information for forensic age estimation [6, 8, 15].

Despite their widespread use, conventional stage-based approaches assign individuals to discrete maturation phases and relate these stages to mean or minimum ages. While such methods offer anthropological interpretability, they implicitly treat skeletal maturation as a stepwise process and inadequately reflect its continuous, non-linear, and probabilistic nature [4, 19]. This limitation becomes particularly problematic near legal age thresholds, where biological variability is greatest and categorical conclusions are least defensible [20, 21].

Recent methodological advances have increasingly framed skeletal maturation as a dynamic biological process rather than a sequence of isolated stages. Non-linear growth models, such as the Gompertz function, provide a biologically plausible framework for capturing asymmetric maturation patterns characterised by rapid adolescent progression followed by gradual deceleration toward skeletal maturity [21–26]. In parallel, probabilistic age modelling has gained prominence in forensic practice by allowing explicit quantification of uncertainty and direct estimation of the likelihood of exceeding legally relevant age thresholds, rather than reliance on single-point estimates [4, 19–21].

Although machine learning approaches have been proposed as powerful tools for forensic age estimation, their added value remains debated in settings where predictor variables are few in number and already biologically interpretable, such as ordinal maturation stages of specific skeletal regions [27–31]. In such contexts, methodological sophistication should primarily enhance the handling of non-linearity and uncertainty rather than introduce opaque prediction mechanisms that may complicate forensic interpretation.

Against this background, the present study investigates the developmental relationship between cervical vertebral maturation (C2–C4) and spheno-occipital synchondrosis ossification in a large forensic CT cohort aged 10 to 28 years. By integrating established ordinal staging systems with non-linear growth modelling and probabilistic age-threshold analysis, this study aims to (i) characterise the relative timing and variability of vertebral and cranial base maturation, (ii) model skeletal development as a continuous biological process, and (iii) provide transparent probability estimates for legally relevant age thresholds.

By prioritising biological plausibility and explicit uncertainty management, this work seeks to enhance the forensic applicability of radiological age estimation in legally sensitive contexts.

## Materials and methods

### Study design, setting, and population

This retrospective cross-sectional study was conducted at Tepecik Training and Research Hospital, Turkey, using medical records and computed tomography (CT) examinations acquired between 2015 and 2020. CT images obtained for clinical indications, predominantly trauma-related assessments, were retrospectively reviewed.

The study population comprised individuals aged 10.0 to 28.0 years, including both males and females. This age range was selected to encompass late childhood, adolescence, and early adulthood, corresponding to the developmental interval most relevant for forensic age assessment. Ethics approval for the study was obtained from the Manisa Celal Bayar University Ethics Committee (Approval No: 1191392/2025). Cases were eligible for inclusion if CT examinations provided adequate visualisation of both the cervical vertebrae (C2–C4) and the spheno-occipital synchondrosis (SOS), allowing reliable assessment of skeletal maturation.Exclusion criteria were defined as: Any pathological condition affecting the cervical vertebrae or cranial base, including tumours, fractures, infections, or surgical fixation, Presence of neoplastic disorders involving the relevant anatomical regions, CT examinations with significant motion artefacts or insufficient image quality precluding accurate evaluation.Only one CT examination per individual was included in the analysis to avoid repeated-measures bias.

The final study sample consisted of 986 individuals, including 621 males and 365 females. The sex distribution reflects the composition of the hospital imaging database during the study period.

For descriptive purposes, individuals were grouped into 1-year age cohorts. For example, individuals classified as age 10 corresponded to a chronological age range of 10.00 to 10.99 years based on date of birth. Sample sizes across age cohorts ranged from 17 to 63 individuals, resulting in a relatively even age distribution across the study population.

Chronological age was calculated as decimal age (in years) by subtracting the date of birth from the CT examination date. Age was treated as a continuous variable in all statistical analyses. For specific forensic applications, age was additionally evaluated in relation to predefined legal thresholds (≥ 14, ≥ 16, and ≥ 18 years), without dichotomisation during modelling.

All CT examinations were performed using a 128-row multidetector CT scanner (Siemens Medical Solutions, Erlangen, Germany). Cervical spine imaging was acquired with axial thin slices of 1.0 mm, followed by sagittal reconstructions.

Images were reconstructed using a standard bone kernel in both axial and sagittal planes. Scan parameters included a tube voltage of 120 kV and a tube current of 120 mAs. All examinations were performed without intravenous contrast administration.

Only CT studies meeting predefined image quality criteria—adequate spatial resolution, absence of motion artefact, and complete coverage of the cervical spine and cranial base—were included in the final analysis.

### Skeletal maturation assessment

Cervical vertebral maturation (C2–C4).

**Fig. 1:**
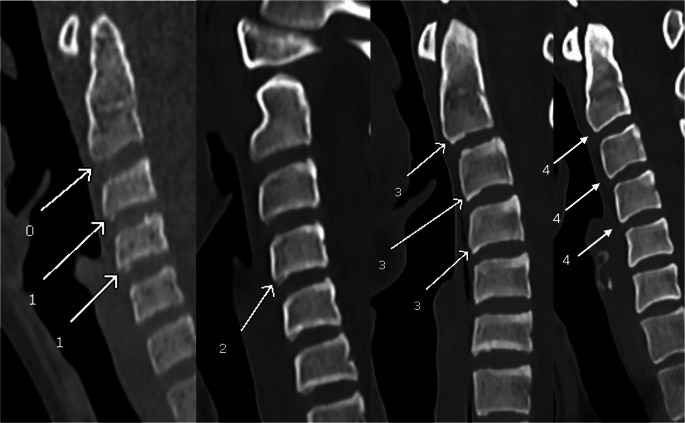
CT-based staging of cervical vertebral ring apophysis maturation (C2–C4) with arrow-guided landmarks. Representative sagittal CT images illustrate the five-stage classification (Stages 0–4) of cervical vertebral ring apophysis maturation at levels C2–C4. Arrows indicate the key morphological features used for stage assignment at each vertebral level

Maturation of the anterior inferior vertebral ring apophysis of the cervical vertebrae C2, C3, and C4 was assessed using the ordinal staging system described by Uys et al., adapted for computed tomography. Each vertebra was scored independently on a five-stage scale (Fig. 1) based on clearly defined morphological criteria [6, 15].

- Stage 0: No ossification of the vertebral ring apophysis is visible. The inferior borders of all cervical vertebrae appear flat or show slight concavity at the inferior borders of C2 and C3. The superior borders taper from posterior to anterior.
- Stage 1: Initial ossification of the apophysis is present. The ossification centre is visible as a small hyperdense area at the anterior margin of the vertebral body, without any union to the inferior border.
- Stage 2: Partial union of the apophysis with the inferior vertebral body is observed at the posterior end of the ossification centre. A hypodense line remains anteriorly between the ossification centre and the vertebral body.
- Stage 3: Complete union of the apophysis has occurred; however, a residual notch or indentation between the apophysis and the inferior vertebral body is still visible.
- Stage 4: Complete fusion is present with a smooth and continuous cortical margin, indicating full maturation of the apophysis.

Assessments were performed on axial and sagittal reconstructions using bone window settings to ensure optimal visualisation of the anterior inferior vertebral margins.

### Spheno-occipital synchondrosis (SOS)

**Fig. 2:**
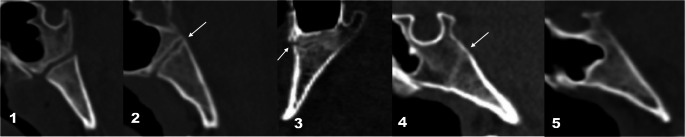
CT-based staging of spheno-occipital synchondrosis (SOS) ossification with arrow-guided landmarks. Representative sagittal CT images demonstrate the five-stage classification of SOS ossification according to the method adapted from Bassed et al. [18]. Arrows indicate the key morphological features used for stage assignment

The degree of ossification of the spheno-occipital synchondrosis (SOS) was evaluated using a five-stage CT-based classification system derived from the method described by Bassed et al. [18]. Each CT examination was assigned to one of the following stages (Fig. 2):

- Stage 1: The synchondrosis is completely open along its entire length.
- Stage 2: Fusion is observed at the superior border of the synchondrosis, while the remainder remains open.
- Stage 3: Approximately half of the length of the synchondrosis is closed.
- Stage 4: The synchondrosis is completely fused; however, a thin horizontal line consistent with an epiphyseal scar remains visible.
- Stage 5: Complete fusion is present with no visible epiphyseal scar.

SOS staging was performed on midline sagittal images, with additional multiplanar reconstructions reviewed as necessary to confirm stage assignment.

All CT images were evaluated by observers experienced in musculoskeletal radiology and forensic medicine. Image assessments were conducted under standardised viewing conditions. Observers were blinded to chronological age and sex at the time of evaluation.

To assess observer reliability, a subset of 55 CT examinations was randomly selected. These cases were re-evaluated two weeks after the initial assessment by the primary observer and independently assessed by a second observer. Intra- and inter-observer agreement for both cervical vertebral staging and SOS staging was evaluated using Cohen’s κ statistic.

Weighted κ values and overall agreement rates were calculated. The strength of agreement was interpreted according to the criteria proposed by Altman [32] where κ < 0.20 indicates poor agreement, κ = 0.21–0.40 fair agreement, κ = 0.41–0.60 moderate agreement, κ = 0.61–0.80 good agreement, and κ = 0.81–1.00 very good agreement.

### Statistical analysis

All statistical analyses were performed with age treated as a continuous variable. Analyses were conducted using R (version ≥ 4.2.0) and Python (version ≥ 3.10) within reproducible computing environments. Descriptive statistics were used to summarise age distributions and stage frequencies for cervical vertebral maturation (C2–C4) and spheno-occipital synchondrosis (SOS) ossification.

### Descriptive and stage-based analyses

Stage frequencies were calculated separately for each cervical vertebra (C2, C3, and C4) and for SOS. For descriptive purposes, maturation stages were examined across one-year age cohorts. No assumptions of normality were imposed on age distributions within stages.

Descriptive analyses and graphical summaries were performed in R, using the packages base, stats, dplyr, tidyr, and ggplot2.

### Transition analysis

To characterise the age-related progression of skeletal maturation, transition analysis was performed to estimate the age ranges over which transitions between successive maturation stages occurred. For each stage transition, the probability of having reached a given stage was examined as a function of chronological age, allowing identification of age intervals associated with increasing likelihood of stage advancement.Transition probabilities and age-dependent stage attainment curves were estimated using custom routines implemented in R, primarily relying on stats, splines, and boot, without imposing parametric assumptions on the underlying age distributions. This approach provided a descriptive framework for assessing the temporal ordering and overlap of maturation stages without assuming linear progression.

### Non-linear growth modelling

Non-linear growth modelling was applied to describe the overall maturation trajectories of cervical vertebrae and SOS across age. A Gompertz growth function was selected due to its suitability for modelling biological processes characterised by an initial acceleration phase followed by asymptotic behaviour.Separate models were fitted for cervical vertebral maturation and SOS ossification using non-linear least squares and optimisation routines implemented in R, primarily through the packages nls, minpack.lm, and optimx. Model parameters describing the growth rate, inflection point, and asymptotic behaviour were estimated, and corresponding 95% confidence intervals were derived using profile likelihood and bootstrap resampling implemented via stats and boot. The Gompertz models were used as descriptive tools to capture non-linear maturation patterns and to facilitate comparison of vertebral and cranial base development across age.

### Probabilistic age modelling and threshold estimation

Probabilistic age modelling was performed to estimate the conditional probability of an individual being at or above predefined legal age thresholds (≥ 14, ≥ 16, and ≥ 18 years), given observed skeletal maturation stages.For each maturation stage and selected combinations of stages, empirical conditional age distributions were derived using Python, with numerical and scientific computations performed using NumPy and SciPy, and data handling facilitated by pandas. These distributions were normalised across the observed age range to obtain posterior age probability profiles. From these profiles, the probability of exceeding each legal threshold was calculated as the proportion of the conditional distribution lying at or above the threshold of interest.This probabilistic framework allowed explicit quantification of uncertainty associated with age estimation and avoided categorical classification based on single-point estimates.

### Machine learning analyses

Machine learning analyses were conducted for comparative purposes only, to assess consistency with probabilistic modelling results. Given the limited number of biologically interpretable predictors, machine learning was not used as the primary analytical framework.

Exploratory supervised learning models were implemented in Python using scikit-learn, including routines for model training, cross-validation, and performance evaluation. Details regarding model architectures, validation procedures, and performance metrics are provided in the Tables 7 and 8.

### Sex-related analyses

Sex was not included as a stratification variable in the primary modelling, as the main objective of the study was to characterise pooled non-linear and probabilistic relationships between skeletal maturation and chronological age at legally relevant thresholds. Nevertheless, sex-related differences were explored using non-parametric statistical tests to evaluate their potential influence on maturation timing. Median ages at each maturation stage were compared between males and females using the Mann–Whitney U test, and differences in stage distributions were assessed using chi-square tests. In addition, sex-related effects were examined across predefined age groups to evaluate potential age-dependent patterns.

### Statistical significance and reporting

All results are reported with appropriate measures of uncertainty. Model parameters are presented together with 95% confidence intervals, and probability estimates are reported as conditional probabilities.

## Results

### Study population

After exclusion of individuals younger than 10 years, a total of 986 cases were included in the final analysis.The study cohort comprised 621 males (63.0%) and 365 females (37.0%). Chronological age ranged from 10.01 to 28.21 years, with a mean age of 17.83 ± 4.20 years and a median age of 17.93 years. The distribution of individuals younger than 18 years (498 cases; 50.5%) and those aged 18 years or older (488 cases; 49.5%) was approximately balanced (Table 1).

**Table 1:** Demographic characteristics of the study cohort

Variable	Total (*n* = 986)	Females (*n* = 365)	Males (*n* = 621)
Mean age, years ± SD	17.83 ± 4.20	17.82 ± 4.03	17.83 ± 4.30
Median age, years	17.93	17.94	17.92
Age range, years	10.01–28.21	10.01–25.01	10.02–28.21
< 18 years, n (%)	498 (50.5%)	185 (50.7%)	313 (50.4%)
≥ 18 years, n (%)	488 (49.5%)	180 (49.3%)	308 (49.6%)

### Observer agreements

Intraobserver and interobserver agreements in evaluating C2-4 and SOS were calculated separately. The intraobserver agreement for the C2 vertebrae was κ = 0.981, and the interobserver reliability was κ = 0.934. The intraobserver agreement for C3 and C4 were κ = 0.980 and 0.972 respectively, and the interobserver reliabilities were κ = 0.972 and 0.934. For the SOS, The intraobserver agreement was κ = 0.984, and the interobserver reliabilities were κ = 0.980. Thus, both intraobserver and interobserver evaluation showed very good repeatability and consistency of the method.

### Distribution of cervical vertebral maturation stages

The distribution of maturation stages for the anterior inferior vertebral ring apophysis of C2, C3, and C4 demonstrated a clear age-related progression (Table 2). Across all vertebral levels, lower stages (Stages 0–1) predominated in younger age cohorts, whereas advanced stages (Stages 3–4) became increasingly prevalent with advancing age.

**Table 2:** Distribution of cervical vertebral maturation stages (C2–C4)

Stage	C2 *n* (%)	C3 *n* (%)	C4 *n* (%)
**0**	86 (8.7)	77 (7.8)	61 (6.2)
**1**	76 (7.7)	81 (8.2)	98 (9.9)
**2**	57 (5.8)	87 (8.8)	95 (9.6)
**3**	147 (14.9)	173 (17.6)	202 (20.5)
**4**	620 (62.9)	568 (57.6)	530 (53.8)
**Total**	986 (100)	986 (100)	986 (100)

**Fig. 3:**
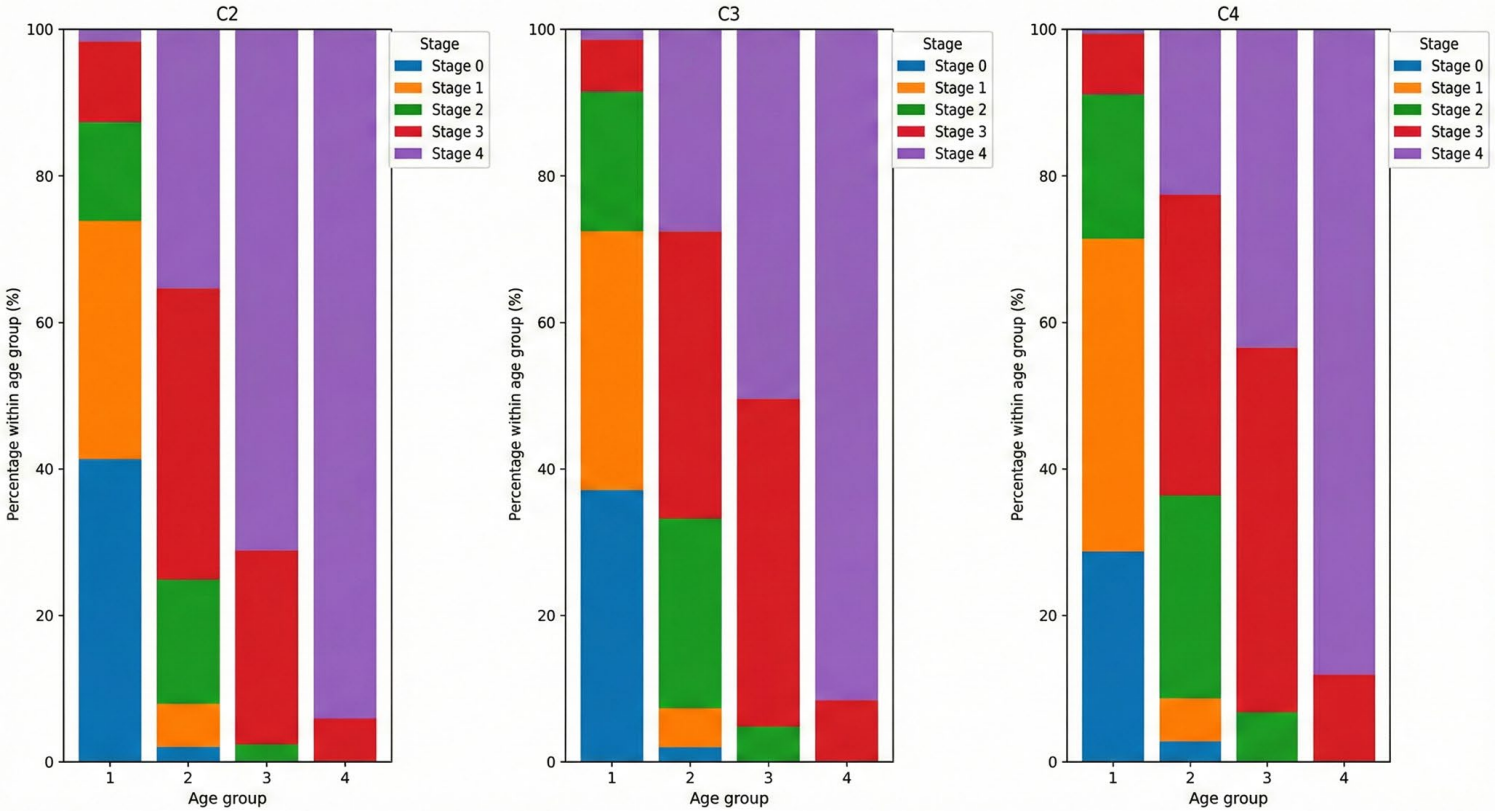
Age-stratified distribution of cervical vertebral maturation stages (C2–C4). Stacked bar charts show the percentage distribution of maturation stages (Stages 0–4) for the anterior inferior vertebral ring apophysis of C2 (Panel A), C3 (Panel B), and C4 (Panel C) across one-year age cohorts (≥ 10 years). A progressive shift toward advanced stages with increasing age is observed for all three vertebrae. Complete fusion (Stage 4) appears earliest and most frequently in C2, followed by C3 and C4, reflecting a cranio-caudal gradient in cervical vertebral maturation

Complete fusion (Stage 4) was observed most frequently in C2 (62.9%), followed by C3 (57.6%) and C4 (53.8%) (Table 2). Age-stratified visualisation further demonstrated that Stage 4 appeared earlier and more prominently in C2, with progressively delayed predominance in C3 and C4 (Fig. 3).

### Distribution of spheno-occipital synchondrosis ossification stages

Of the 986 individuals, approximately 976 cases were suitable for assessment of spheno-occipital synchondrosis (SOS). The distribution of SOS stages revealed a broader and more heterogeneous age pattern compared with cervical vertebral maturation (Table 3).

**Table 3:** Distribution of spheno-occipital synchondrosis (SOS) stages

SOS stage	*n*	%
Stage 1	121	12.3
Stage 2	91	9.2
Stage 3	53	5.4
Stage 4	336	34.1
Stage 5 (complete fusion)	385	39.0
**Total**	**986**	**100**

**Fig. 4:**
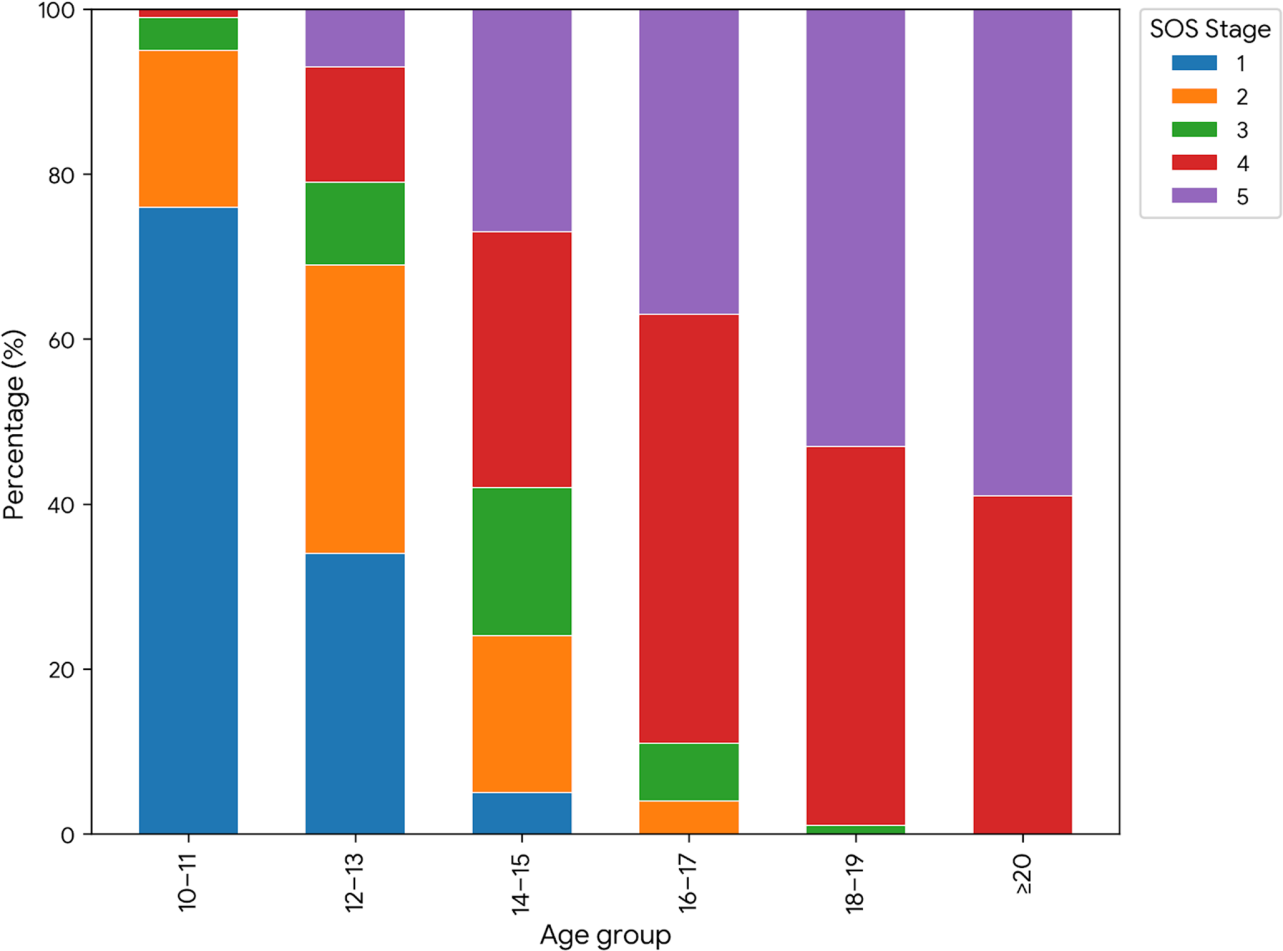
Age-stratified distribution of spheno-occipital synchondrosis (SOS) ossification stages. Stacked bar charts show the percentage distribution of SOS ossification stages (Stages 1–5) across one-year age cohorts (≥ 10 years). Early stages predominate in younger age groups, whereas complete fusion (Stage 5) becomes more frequent in late adolescence and early adulthood. The broad overlap between advanced stages highlights the prolonged and variable nature of SOS maturation

Early stages (Stages 1–2) were primarily observed in younger age cohorts, whereas advanced stages (Stages 4–5) became increasingly common in later adolescence and early adulthood. Complete fusion without a visible epiphyseal scar (Stage 5) was observed in **approximately 39.0%** of evaluable cases (Table 2). Age-stratified analysis illustrated substantial overlap between advanced SOS stages across multiple age cohorts, reflecting prolonged and variable maturation (Fig. 4).

### Transition analysis

Transition analysis demonstrated overlapping age ranges for successive maturation stages in both cervical vertebrae and SOS. Transitions to advanced stages occurred over relatively narrow age intervals for cervical vertebrae, whereas SOS transitions extended over wider age ranges, particularly for the transition to complete fusion (Table 4).

**Table 4:** Regression analysis of the association between chronological age and skeletal maturation stages

Predictor (stage)	β coefficient (years per stage)	Standard Error (SE)	95% Confidence Interval	*p*-value
**C2 stage**	1.42	0.04	1.34–1.50	< 0.001
**C3 stage**	1.36	0.05	1.26–1.46	< 0.001
**C4 stage**	1.28	0.05	1.18–1.38	< 0.001
**SOS stage**	0.91	0.04	0.83–0.99	< 0.001

### Association between chronological age and skeletal maturation

Linear regression analysis confirmed significant associations between chronological age and maturation stage for all skeletal indicators examined (Table 4). Cervical vertebral stages showed stronger associations with age, with regression coefficients of 1.42 years per stage for C2, 1.36 for C3, and 1.28 for C4. In contrast, SOS stage demonstrated a lower coefficient (0.91 years per stage), consistent with a slower and more variable maturation process (Table 4).

### Non-linear maturation trajectories

**Fig. 5:**
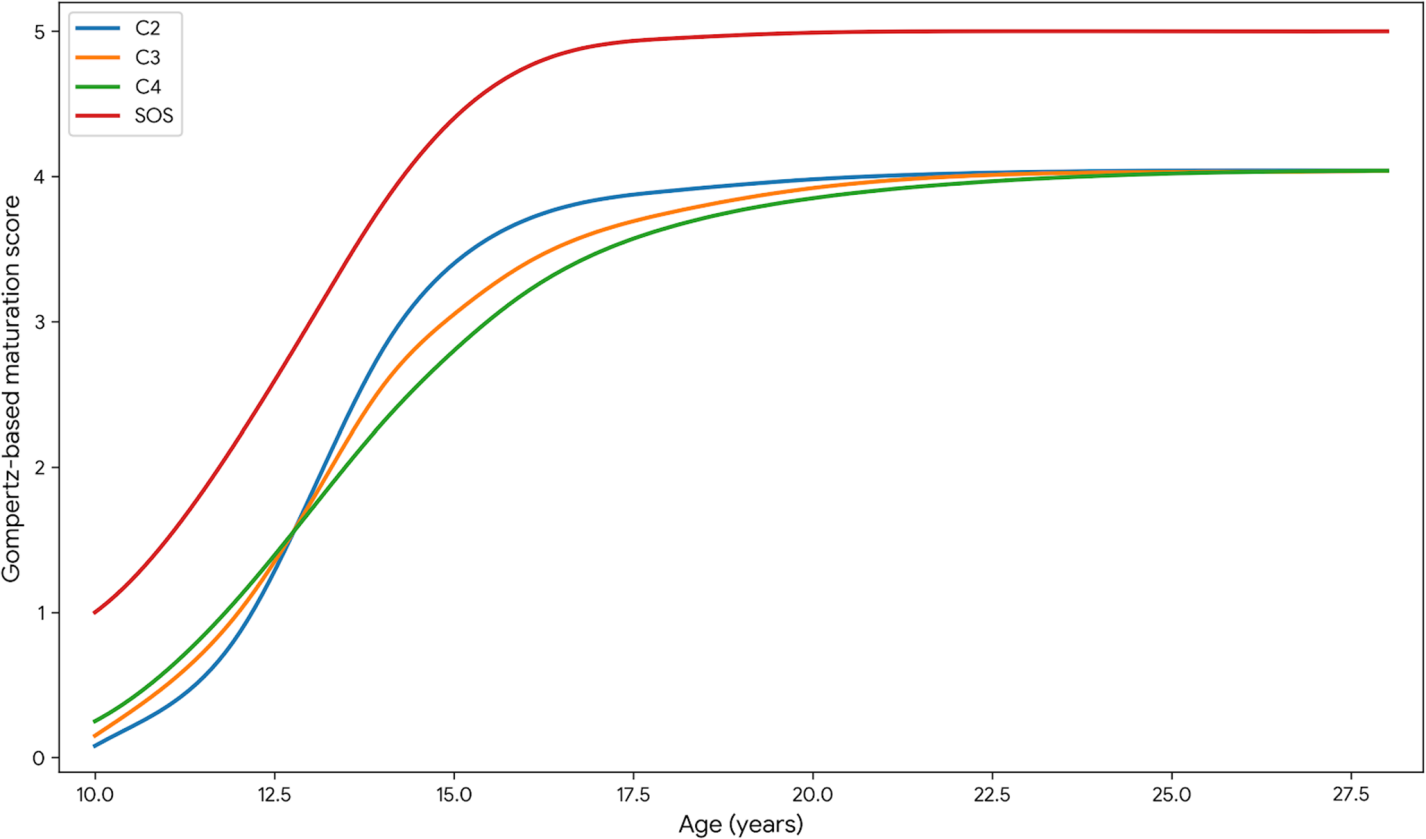
Non-linear skeletal maturation trajectories based on Gompertz growth modelling. Gompertz curves illustrate age-related maturation patterns of cervical vertebrae (C2–C4) and the spheno-occipital synchondrosis (SOS). Cervical vertebral maturation demonstrates earlier inflection points and steeper growth compared with SOS, which shows delayed and more gradual progression toward complete ossification. These trajectories highlight differences in the timing and variability of vertebral and cranial base maturation

Non-linear Gompertz growth models provided an adequate description of maturation trajectories for both cervical vertebrae and SOS (Table 5). Cervical vertebral maturation exhibited earlier inflection points (approximately 15.8–16.6 years) and steeper growth compared with SOS, which demonstrated a delayed inflection point (approximately 18.9 years) and a more gradual progression toward asymptotic maturity (Table 5). These differences are visually apparent in the fitted Gompertz curves (Fig. 5).

**Table 5:** Gompertz model parameters for skeletal maturation

Anatomical site	Growth rate (b)	Inflection age (years)	Asymptote	Model fit (*R*²/pseudo-*R*²)
**C2**	High	~ 15.8	4.0	0.82
**C3**	High	~ 16.2	4.0	0.80
**C4**	Moderate	~ 16.6	4.0	0.78
**SOS**	Low–moderate	~ 18.9	5.0	0.74

### Probabilistic age threshold estimation

Conditional probability analysis revealed distinct differences in the likelihood of exceeding legally relevant age thresholds based on skeletal maturation indicators (Table 6). Advanced cervical vertebral maturation (Stage 4) was associated with high probabilities of being at or above 14, 16, and 18 years, with probabilities decreasing in a stepwise manner from C2 to C4.

**Table 6:** Conditional probabilities of exceeding legal age thresholds based on skeletal maturation

Skeletal indicator	*P*(age ≥ 14 years)	*P*(age ≥ 16 years)	*P*(age ≥ 18 years)
**C2 – Stage 4**	0.98	0.94	0.88
**C3 – Stage 4**	0.97	0.92	0.84
**C4 – Stage 4**	0.95	0.90	0.81
**SOS – Stage 5**	0.93	0.86	0.72
**Combined configuration (C2–C4 + SOS)**	0.99	0.96	0.91

**Fig. 6:**
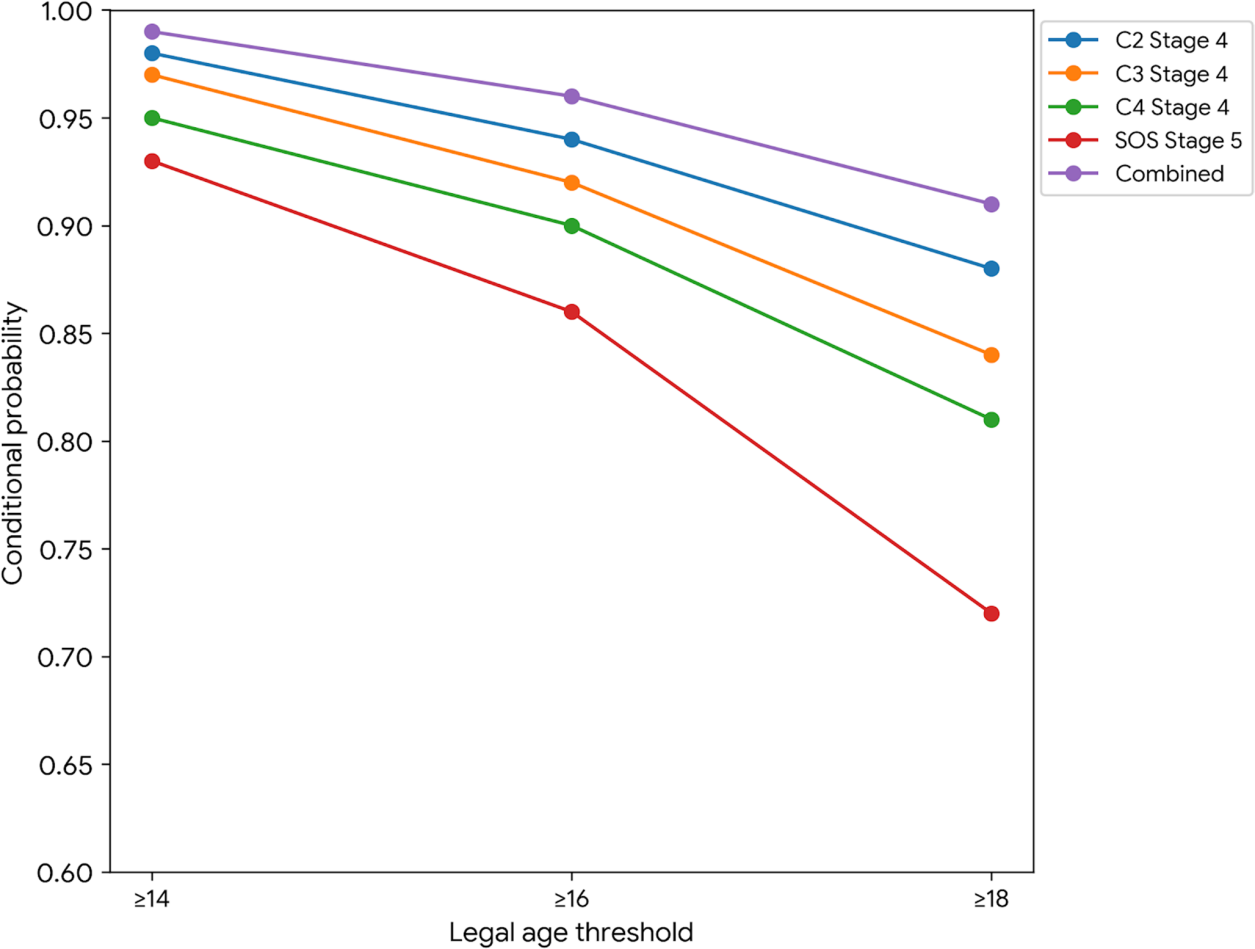
Conditional probability of exceeding legally relevant age thresholds based on skeletal maturation. Line plots depict the conditional probability of being at or above 14, 16, and 18 years of age given specific skeletal maturation indicators. Cervical vertebral maturation (Stage 4) demonstrates consistently higher probabilities across thresholds compared with complete spheno-occipital synchondrosis fusion (Stage 5). The combined configuration of cervical vertebrae and SOS yields the highest conditional probabilities at all thresholds, indicating reduced uncertainty when multiple skeletal indicators are considered

Complete SOS fusion (Stage 5) was associated with lower conditional probabilities across all thresholds, particularly for the ≥ 18-year threshold (*P* = 0.72). The combined configuration incorporating both cervical vertebral maturation and SOS yielded the highest conditional probabilities at all thresholds (*P* ≥ 0.99 for ≥ 14 years; *P* = 0.96 for ≥ 16 years; *P* = 0.91 for ≥ 18 years), indicating reduced uncertainty when multiple skeletal indicators were considered (Table 6; Fig. 6). Despite statistically detectable sex-related differences in maturation timing, these differences did not persist uniformly across all age groups and were markedly attenuated at ages relevant to legal decision-making. Consequently, sex-specific stratification did not provide a clear advantage for probabilistic inference at legally relevant age thresholds, supporting the use of pooled models for the primary analyses.

### Machine learning analyses

Machine learning analyses were conducted for comparative purposes using cervical vertebral and SOS staging variables as predictors. Overall, classification performance at legally relevant age thresholds followed patterns consistent with probabilistic modelling results, with cervical vertebral maturation providing higher discrimination than SOS alone. However, machine learning models did not yield additional biologically interpretable information beyond that provided by non-linear and probabilistic approaches. For this reason, machine learning results were not used as the primary analytical framework and are presented in detail in the Table 7.

**Table 7:** Sex-related differences in skeletal maturation stages across cervical vertebrae (C2–C4) and spheno-occipital synchondrosis (SOS)

Anatomical region	Stage	Female median age (years)	Male median age (years)	*p*-value
**C2**	0	10.80	11.19	0.221
	1	11.33	12.51	**0.0019**
	2	12.64	14.51	**< 0.001**
	3	15.00	15.75	**0.010**
	4	19.46	20.75	**< 0.001**
**C3**	0	10.84	11.07	0.244
	1	11.30	12.25	**0.024**
	2	12.74	14.73	**< 0.001**
	3	15.54	16.52	**< 0.001**
	4	19.58	21.04	**< 0.001**
**C4**	0	10.88	11.39	0.326
	1	11.27	11.93	**0.006**
	2	13.00	14.73	**< 0.001**
	3	15.71	16.72	**0.005**
	4	19.87	21.30	**< 0.001**
**SOS**	1	10.88	11.46	0.075
	2	11.47	13.99	**< 0.001**
	3	13.87	15.47	**< 0.001**
	4	18.33	19.44	**< 0.001**
	5	19.33	20.96	**< 0.001**

### Sex-related differences

Sex-related differences in skeletal maturation were observed across both cervical vertebral maturation (C2–C4) and spheno-occipital synchondrosis (SOS). Overall, males exhibited a delayed maturation pattern compared with females, as reflected by significantly higher median ages at corresponding maturation stages for most vertebral levels and SOS stages.

When analyses were stratified by age groups, these sex-related differences were most pronounced in the younger and intermediate age groups and progressively diminished with advancing age. In the oldest age group, no statistically significant sex differences were observed for either C2–C4 or SOS maturation stages, indicating substantial biological overlap between sexes at later stages of skeletal maturation.

When cervical vertebral maturation and spheno-occipital synchondrosis were evaluated in combination, the conditional probabilities of having reached legally relevant age thresholds increased substantially compared with single indicators, while sex-related differences were attenuated, particularly at the ≥ 16- and ≥ 18-year thresholds (Table 8).

**Table 8:** Combined method: sex-specific probabilities for legally relevant age thresholds. Conditional probabilities based on combined cervical vertebral maturation (C2–C4) and SOS stages

Cmax	SOS stage	Sex	*P*(age ≥ 14)	*P*(age ≥ 16)	*P*(age ≥ 18)
≥ 3	≥ 3	Female	~ 0.85	~ 0.55	~ 0.30
≥ 3	≥ 3	Male	~ 0.97	~ 0.70	~ 0.32
≥ 4	≥ 3	Female	~ 0.95	~ 0.75	~ 0.55
≥ 4	≥ 3	Male	~ 0.99	~ 0.90	~ 0.70
≥ 4	≥ 4	Female	~ 0.98	~ 0.82	~ 0.60
≥ 4	≥ 4	Male	~ 1.00	~ 0.94	~ 0.78
≥ 4	5	Female	~ 0.99	~ 0.85	~ 0.65
≥ 4	5	Male	~ 1.00	~ 0.96	~ 0.82

Cmax = maksimum C2–C4 stages

Despite these observed sex-related differences, sex stratification did not consistently improve discrimination or reduce uncertainty at legally relevant age thresholds; therefore, sex-specific modelling was not adopted as the primary analytical framework.

## Discussion

This study proposes a multi-layered analytical framework for forensic age assessment [17, 28, 29] by jointly evaluating cervical vertebral maturation (C2–C4) and spheno-occipital synchondrosis (SOS) ossification [6, 7] in a large CT-based forensic cohort aged 10 years and older. By integrating established ordinal staging systems [1, 9] with non-linear growth modelling and probabilistic age-threshold analyses [22, 23, 26], the approach allows biological continuity of skeletal maturation and inter-individual variability to be more explicitly characterised [25].

Cervical vertebrae mature earlier and within a narrower age range than SOS [7, 8, 16]. Complete fusion (Stage 4) occurs most frequently and at younger ages in C2, followed sequentially by C3 and C4, consistent with the expected cranio-caudal maturation gradient of the cervical spine [6, 11, 15]. Accordingly, cervical vertebrae represent relatively age-sensitive indicators, particularly during mid-adolescence [9].

n contrast, SOS ossification exhibits a broader and more heterogeneous age distribution [7, 16, 18]. Although advanced SOS stages increase with age, the substantial overlap between Stages 4 and 5 indicates a prolonged and variable maturation trajectory of the cranial base [8, 16, 25]. Consequently, when considered in isolation, SOS provides limited discriminative value near legally relevant age thresholds [1, 28, 29].

Previous forensic imaging studies have identified both cervical vertebral maturation and spheno-occipital synchondrosis ossification as relevant indicators of skeletal development during adolescence and early adulthood [6–8, 28]. CT- and MRI-based investigations consistently describe a cranio-caudal maturation pattern within the cervical spine, with C2 reaching advanced stages earlier than lower cervical levels [6, 11, 15]. The sequential pattern observed in the present cohort is compatible with these findings, and the relatively narrow age dispersion of advanced cervical stages further supports their relevance in age-sensitive forensic contexts [1, 5, 9].

With regard to the spheno-occipital synchondrosis, prior studies have repeatedly emphasised its prolonged and variable maturation, particularly during late adolescence [7, 16, 18]. Overlap between advanced stages, especially between incomplete and complete fusion, has been reported across populations and imaging modalities [8, 16, 17]. The present findings align with this pattern and reinforce the association of isolated SOS assessment with substantial uncertainty near legally relevant age thresholds [1, 3, 28]. Importantly, the present study extends previous work by explicitly contextualising this variability within a probabilistic framework [23, 28, 29].

Importantly, many earlier investigations have focused on single anatomical regions [6, 7, 15, 16] or relied primarily on categorical interpretations of maturation stages [1, 9, 12]. By contrast, the present study integrates multiple skeletal indicators within a unified analytical framework [17, 20, 28] that incorporates non-linear modelling and conditional probability estimation [22, 23, 26]. Rather than replacing established methods, this approach contextualises them within a probabilistic structure [24, 29] that more explicitly reflects biological variability [25] and supports proportionate medico-legal decision-making [4, 5, 31].

Gompertz growth modelling provides a functional description of the non-linear nature of skeletal maturation [26, 32]. Cervical vertebrae demonstrate earlier inflection points and steeper growth trajectories, whereas SOS shows delayed inflection and more gradual progression toward a plateau (Table 5; Fig. 5) [6, 7, 16]. This divergence is consistent with biological knowledge of the distinct developmental roles of these structures [2, 31]. Accordingly, non-linear modelling complements rather than replaces classical staging systems by expressing age-related behaviour within a continuous framework while preserving anthropological interpretability [22, 28, 29].

Regression analyses demonstrate statistically meaningful associations between chronological age and all examined skeletal indicators, although association strength varies by anatomical region (Table 4) [6, 7, 30]. Cervical vertebrae exhibit higher regression coefficients than SOS, indicating more consistent age-related progression [6, 9, 15], whereas the lower coefficient observed for SOS is compatible with increased biological variability, particularly during late adolescence [8, 16, 18]. These differences help explain why single-region approaches may be One of the most important contributions of this study lies in the probabilistic interpretation of legal age thresholds [1, 23, 29]. Conditional probability analyses show that advanced cervical vertebral maturation is associated with high probabilities of exceeding the 14-, 16-, and 18-year thresholds, with probabilities decreasing progressively from C2 to C4 (Table 6; Fig. 6) [6, 15, 24]. By contrast, complete SOS fusion is associated with lower probabilities, particularly for the ≥ 18-year threshold [7, 16, 18].

These findings indicate that a single biological event, such as complete SOS ossification, is insufficient as a determinant of legal adulthood when considered in isolation [1, 2, 16]. In contrast, the combined evaluation of cervical vertebrae and SOS yields higher conditional probabilities across all thresholds and a narrower range of uncertainty [17, 28, 29], supporting the value of multi-indicator approaches for uncertainty reduction in medico-legal decision-making [4, 5, 23, 31].

Machine learning analyses were examined for comparative purposes [27, 30]. Although these models reproduced the general pattern of higher discriminative capacity for cervical vertebrae relative to SOS, they did not provide additional biologically interpretable insights beyond those obtained through probabilistic modelling [23, 28, 29]. In contexts with a limited number of predictors and clear biological meaning, increased model complexity appears to offer performance comparable to simpler, interpretable frameworks [22, 26], reinforcing the principle that analytical complexity should remain a means rather than an end in forensic applications [4, 25, 31].

Sex-related differences in skeletal maturation have been consistently reported in the literature and were also observed in the present dataset, particularly during early and mid-adolescence. In line with previous studies, males generally exhibited delayed maturation relative to females [6–18]. However, these differences were strongly age-dependent and diminished substantially during later adolescence and early adulthood.

From a forensic perspective, this age-dependent attenuation limits the practical utility of sex-specific cut-offs for age-threshold assessment. Given the pronounced overlap in maturation stages at later ages, pooled probabilistic models more accurately reflect underlying biological variability and avoid overinterpretation of sex-related differences that do not meaningfully improve decision-making at legally relevant age thresholds.

Although the present study is based on CT data, the analytical framework described here may be transferable to radiation-free imaging modalities in living individuals [1, 5, 31]. In particular, magnetic resonance imaging (MRI) enables assessment of the cervical vertebrae and cranial base without ionising radiation and may facilitate the development of more ethically acceptable age assessment models [8, 28, 30]. When combined with probabilistic and multi-indicator strategies, MRI-based protocols have the potential to support age estimation approaches that explicitly manage uncertainty while addressing ethical considerations in living populations [28, 29].

Overall, the findings support a shift away from categorical age assignments toward probabilistic interpretations that explicitly acknowledge uncertainty [22, 23, 26]. In legal contexts where age thresholds are critical, the presentation of conditional probabilities may facilitate more proportionate and defensible decision-making [1, 4, 31].

Several limitations should be acknowledged. The cross-sectional design precludes direct observation of individual maturation trajectories, and the observed patterns reflect population-level associations. In addition, the use of a single-centre cohort may limit generalisability. Future studies incorporating more diverse populations and MRI-based protocols may further refine and extend the probabilistic framework presented here. Although sex-related differences were observed, this study did not aim to derive sex-specific reference models. Future work may explore whether incorporating sex as a covariate improves probabilistic performance in specific populations; however, the present findings suggest that such stratification is of limited value at later ages.

In conclusion, cervical vertebral maturation follows an earlier and less variable trajectory than SOS ossification. The combined application of non-linear growth modelling and probabilistic age-threshold analysis provides a transparent and manageable representation of uncertainty in forensic age assessment. Evaluating multiple skeletal indicators together may offer a more balanced and legally defensible framework than single-indicator approaches, and further integration of additional anatomical regions may strengthen the legal relevance of probabilistic age indicators.
